# Does Size Matter? The Case of Piezoresistive Properties of Carbon Nanotubes/Elastomer Nanocomposite Synthesized through Mechanochemistry

**DOI:** 10.3390/nano12213741

**Published:** 2022-10-25

**Authors:** Antonio Turco, Anna Grazia Monteduro, Francesco Montagna, Elisabetta Primiceri, Mariaenrica Frigione, Giuseppe Maruccio

**Affiliations:** 1 CNR Nanotec Institute of Nanotechnology, Via Monteroni, 73100 Lecce, Italy; 2 Omnics Research Group, Department of Mathematics and Physics “Ennio De Giorgi”, University of Salento, Via per Monteroni, 73100 Lecce, Italy; 3 Department of Innovation Engineering, University of Salento, Prov.le Lecce-Monteroni, 73100 Lecce, Italy

**Keywords:** electrical properties, carbon nanotubes, nano composites, mechanical properties, flexible composites

## Abstract

The growing interest in piezoresistive sensors has favored the development of numerous approaches and materials for their fabrication. Within this framework, carbon nanotubes (CNTs) are often employed. However, CNTs are a heterogeneous material with different morphological characteristics in terms of length and diameter, and, so far, experimental studies have not usually considered the effect of these parameters on the final sensor performances. Here, we observe how, by simply changing the CNTs length in a solvent-free mechanochemistry fabrication method, different porous 3D elastomeric nanocomposites with different electrical and mechanical properties can be obtained. In particular, the use of longer carbon nanotubes allows the synthesis of porous nanocomposites with better mechanical stability and conductivity, and with a nine-times-lower limit of detection (namely 0.2 Pa) when used as a piezoresistive sensor. Moreover, the material prepared with longer carbon nanotubes evidenced a faster recovery of its shape and electrical properties during press/release cycles, thus allowing faster response at different pressures. These results provide evidence as to how CNTs length can be a key aspect in obtaining piezoresistive sensors with better properties.

## 1. Introduction

The future generations of portable and foldable devices such as wearable flexible electronics, medical implants and electronic skins will require the development of highly sensitive, stretchable and low-cost pressure sensors [1,2,3]. For example, an electronic skin could generate signals able to reflect the strength and the location of an external pressure, but a stability of the sensing materials of up to 55% strain is necessary [4]. Under this view, different strategies have been proposed, e.g., relying on transistor [5], piezoelectric [6], capacitive [7] and piezoresistive sensing [8]. Among them, piezoresistive transducers have attracted researcher interest, due to the relative simplicity of signal collection and the possibility of preparing highly flexible materials. The first approaches proposed the use of a polymer streamer with metal layers on the surface. However, these devices were usually able to detect only small strains (~5%) with relatively low sensitivity, due to problems such as the cracking of the materials [9], which can affect the sensor reproducibility and stability and could cause environmental and health concerns [10,11]. Another strategy is to prepare polymeric 2D films mixed with conductive (nano)materials for the fabrication of the sensors. However, the application of these materials is limited, due to the difficulties in dispersing conductive nanomaterials [12], low sensitivity, instability and the impossibility of detecting low pressures. To solve these issues, the synthesis of the polymeric materials in the form of sponges represented a good alternative strategy for their better mechanical stability and electronic properties. Several conductive sponges have been prepared using porous polymers mixed with metallic nanoparticles, nanowires or carbon-based nanomaterials, which must be stably entrapped in the composites to avoid health concerns [11,13,14,15]. Among them, nanomaterials composed of graphitic carbon such as carbon nanotubes and two-dimensional graphene sheets have evidenced better performances in terms of stability and sensitivity [16,17,18,19,20,21]. Different approaches have been proposed to combine these nanomaterials with porous polymers, and the most common strategy is to cover the surface of pre-shaped polymeric sponges through adsorption of the conductive nanomaterials [22,23]. Only recently, we proposed an easy procedure to combine these materials during polymerization steps in order to give a better dispersion of the nanomaterial on both the surface and within the bulk of the polymeric sponges, thus allowing the production of nanocomposites with better stability and sensing performances [20]. In all the piezoresistive sponges, the sensing mechanism is based on the change in resistance signal caused by the variation of the number and shape of contact points between the different conductive (nano)particles in the material. Considering the huge number of dimensions of these nanomaterials, it is evident as an important parameter for obtaining highly a sensitive piezoresistive sensor is represented by the morphological characteristics of the nanomaterials. Consequently, a careful investigation into the effect of the nanomaterial morphology on piezoresistive properties should be performed. In this work, a systematic study on piezoresistive performances of different PDMS/CNTs sponges produced using nanomaterials with the same diameter and different length, was performed. The results evidenced the way in which this aspect can represent a critical parameter in order to tune the performances of the piezoresistive materials such as sensitivity, limit of detection and response time. These findings are useful for obtaining nanocomposites with improved performances, since common reactions (e.g., chemical oxidation) used during piezoresistive sensor fabrication can cause a change in the length of the pristine materials [24].

## 2. Materials and Methods

### 2.1. Chemicals

A polydimethylsiloxane polymerization kit (Sylgard 184) was purchased from Dow Corning. Multiwalled carbon nanotubes (20–30 nm in diameter) synthesized by chemical vapor deposition (CVD) were provided by Nanoamor (https://www.nanoamor.com (accessed on 20 October 2022)). Commercial sugar particles (290 ± 170 μm) were purchased from Co.Pro.B.—Cooperativa Produttori Bieticoli. All the other chemicals were purchased from Sigma Aldrich, and used as received.

### 2.2. Preparation of PDMS/CNTs Conductive Sponge

Commercial sugar microparticles were mixed with 3% of pristine multiwalled carbon nanotubes (MWCNTs) with a tube rotator shaker (Multi Bio RS-24, Biosan, Riga, Latvia; rotation speed = 70 rpm) overnight and without solvents, at room temperature. Two different kinds of MWCNTs with the same diameter but different lengths were separately used to obtain two different nanocomposites. Each mixture was then separately immersed in a solution containing the PDMS prepolymer diluted in hexane at a ratio of 2:3 *w*/*w*. The solid/liquid composites were blended for ten minutes, and the sugar particles with adsorbed MWCNTs were manually separated with a wide mesh sieve. The composites were packed by filling Teflon molds with a defined shape and dimensions. The stamps were then closed with a metal foil, and sealed with screws. The prepared mixtures were cured in an oven at 80 °C for 3 h to accomplish the PDMS polymerization. The metal from the top of the stamp was then removed. The stamps were scratched on their surface to remove the small amount of leaked polymerized mixture, and then immersed in hot water to remove the sugar particles. Finally, two different spongeous materials were detached from the stamps, which were denominated PDMS/CNTs_short_ and PDMS/CNTs_long,_ depending on whether shorter or longer carbon nanotubes were used, respectively. The as obtained 3D porous nanocomposites were used for further characterizations, without other treatments.

### 2.3. Characterization

The morphological characterization of the PDMS/CNTs foam samples was carried out using a scanning electron microscope (SEM, Carl Zeiss Merlin, Oberkochen Germany). SEM images were acquired in top view configuration by using an acceleration voltage of 5 kV, and by employing a secondary electron detector for low magnification and an in-lens detector for high magnification scans. A morphological characterization of CNTs was performed using a transmission electron microscope (TEM, Philips EM208, Amsterdam, Netherland), using an accelerating voltage of 100 kV. The samples were prepared by dropping an aliquot of CNTs dispersed in dimethylformamide onto a TEM grid (200 mesh, Nichel, carbon only).

During piezoresistive measurements the foam (generally produced with a mold of 1.6 × 1.6 × 1 cm) was placed between two cleaned metallic plates, to guarantee electrical contact. A copper wire was welded onto the surface of each plate, and connected to a Keithley 2400 source meter. A current of 100 μA was applied to the sample, and the voltage was recorded in order to evaluate the resistance variation as a function of the compression. The control of the pressure applied to the PDMS/CNTs foams was performed by placing the foam with the planar electrodes between two circular parallel plate tools (50 mm in diameter) installed on a LLOYD LR50K Plus dynamometer with a 100 N load cell. More experimental details are provided in the results and discussion section.

## 3. Preparation of PDMS/CNTs Conductive Sponges

**Figure 1:**
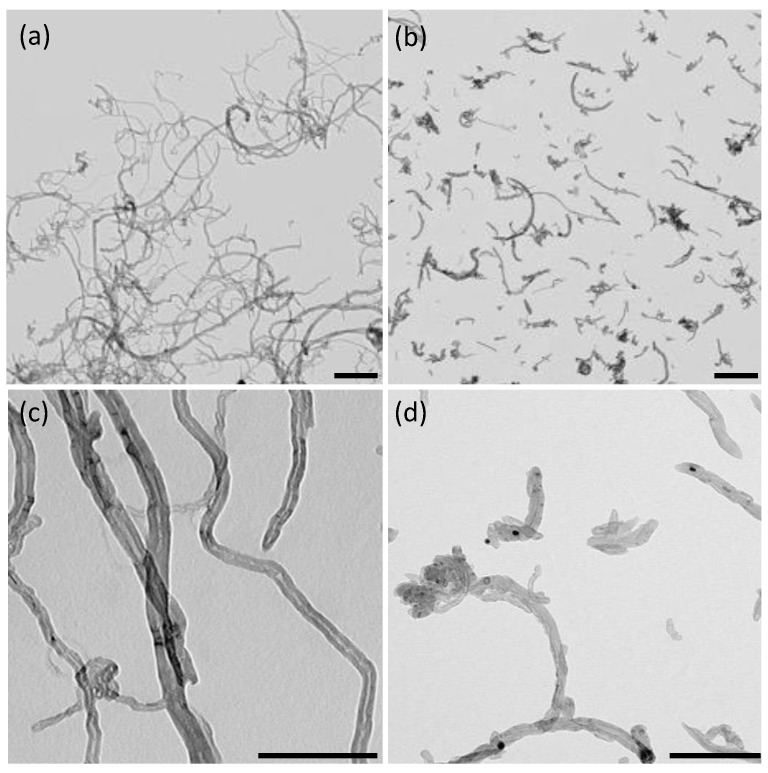
TEM images of CNTs_long_ (**a**,**c**) and CNTs_short_ (**b**,**d**). The scale bar is 500 nm in (**a**,**b**) and 200 nm in (**c**,**d**).

To evaluate the effect of a nanomaterial dimension on the morphological and electrical properties of PDMS/CNTs composite foam, we selected two kinds of multiwalled carbon nanotubes, both synthesized by chemical vapor deposition with the same diameter but with a significant difference in length as observable by TEM (Figure 1). The shorter carbon nanotubes (CNTs_short_) had a length typically between 0.5 and 2 μm (Figure 1b), whilst the longer CNTs (CNTs_long_) were in the range of 10–30 μm (Figure 1a). Both kinds of CNTs did not evidence any significant amount of amorphous carbon and metallic particles (Figure 1c,d). Therefore, we decided to use the nanomaterial in the as-received form, without further purification steps. This allowed us to use a greener fabrication method of the material and avoid possible differences due to different degrees of functionalization, which can be obtained after the purification steps [25].

**Figure 2:**
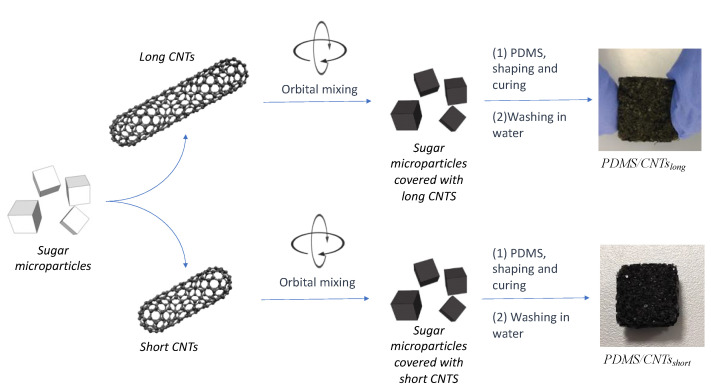
Fabrication scheme of the PDMS/CNTs foams with different carbon nanotube length.

Therefore, to produce spongeous piezoresistive sensors, we used a successful procedure based on a solvent-free mechanochemistry approach [20,26,27,28,29]. This allowed us to avoid the functionalization of the nanomaterials, thus preserving their morphological and electrical properties (Figure 2). In brief, the MWCNTs were mixed overnight in an opportune ratio with the sugar microparticles using a rotatory shaker. During this step, the tangled carbon nanotubes were able to impact with the sugar microparticles, thus addressing the disruption of the MWCNTs bundles, due to the mechanical breaking of π-π interactions. At the same time, the nanomaterial could be homogenously adsorbed onto the glucose crystals surface. After that, the mixtures with the two different MWCNTs were mixed with the same amount of PDMS prepolymer diluted with hexane in the ratio 2:3 *w*/*w*. The as-obtained mixtures were then sieved to remove the polymer in excess, and cured in a closed stamp under constant pressure. Finally, the sugar crystals were dissolved by simply washing the composite in hot water, thus obtaining two sponges with pores of dimensions comparable with the sugar crystals. The two nanocomposites were nominated PDMS/CNTs_short_ and PDMS/CNTs_long,_ depending on the type of MWCNTs used.

**Figure 3:**
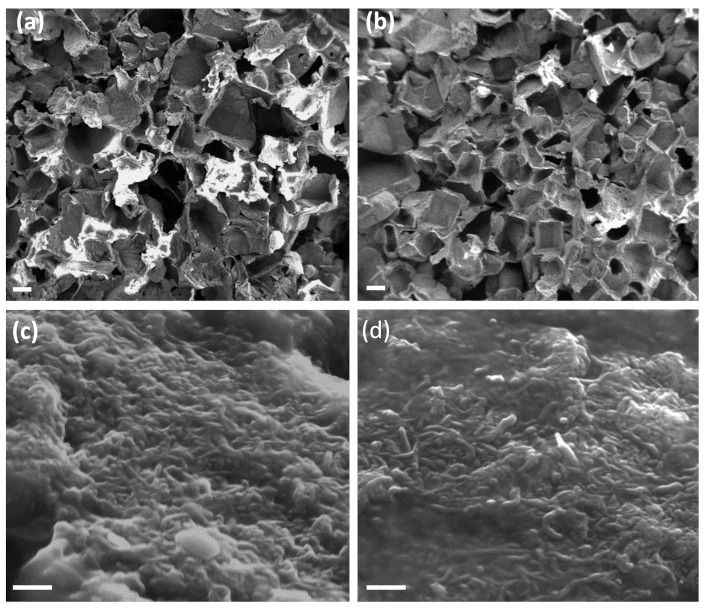
SEM images of PDMS/CNTs_long_ (**a**,**c**) and PDMS/CNTs_short_ (**b**,**d**) foams. The scale bar is 200 μm in (**a**,**b**) and 200 nm in (**c**,**d**).

In both the foams, carbon nanomaterial is well dispersed in the PDMS matrix and on the surface (Figure 3), with a comparable pore distribution (Figure S1), thus suggesting both the parameters are not affected by the kind of MWCNTs used. Due to the synthetic pathway, both the nanocomposites presented an open-pore structure with interconnected cavities; otherwise, sugar crystals would be visible inside the polymeric structure in the SEM images.

**Figure 4:**
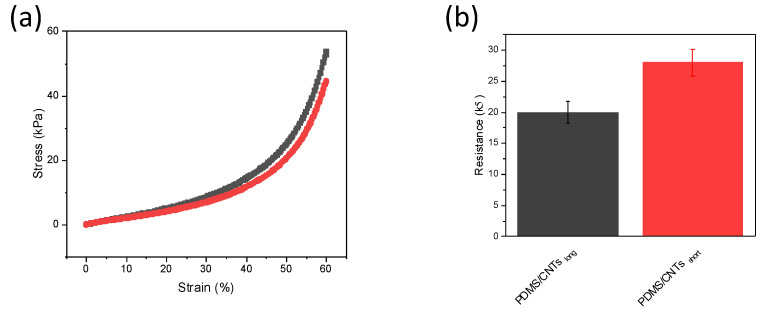
(**a**) Compressive stress–strain curves at 60% strain of PDMS/CNTs_long_ (gray curve) and PDMS/CNTs_short_ (red curve), (**b**) recorded resistance of PDMS/CNTs_long_; PDMS/CNTs_short_ (error bars are calculated on *n* = 3).

The mechanical stability of the two foams at different pressures was evaluated within a range of between 0 and 60% strain (Figure 4a). The stress at the same strain is higher in the foam fabricated with longer carbon nanotubes. This is interesting, since although some theoretical and experimental studies have been performed evaluating the effect of diameter and chirality of single carbon nanotubes on their mechanical strength [30,31,32], no evidence has been reported of the influence of the CNT length on the final properties in nanocomposites when used as fillers. This is important, since numerous reactions used to produce nanocomposites with well-dispersed CNTs (e.g., chemical oxidation), causes a change in the length of the nanomaterial. Therefore, such kind of reactions could not only impair the CNT electronic structure, but can partially compromise the final mechanical stability of the nanocomposites. In addition, the conductivity of the composite material seems to be affected by the length of the nanomaterials (Figure 4b). The analysis of PDMS/CNTs_short_ and PDMS/CNTs_long_ sponges of the same dimensions revealed that the conductivity of the material is higher if longer CNTs are used. This can be explained by the better electron transfer that can be obtained on longer carbon nanotubes. In fact, a lower number of gap junctions between CNTs must be overcome during electronic transfer, thus improving the percolation network and decreasing the overall resistance of the nanocomposite.

Both these aspects suggest the possibility that piezoresistive properties of the two materials can also be affected, depending on the length of the carbon nanomaterial used.

## 4. Piezoresistive Properties of PDMS/CNTs Conductive Sponges

**Figure 5:**
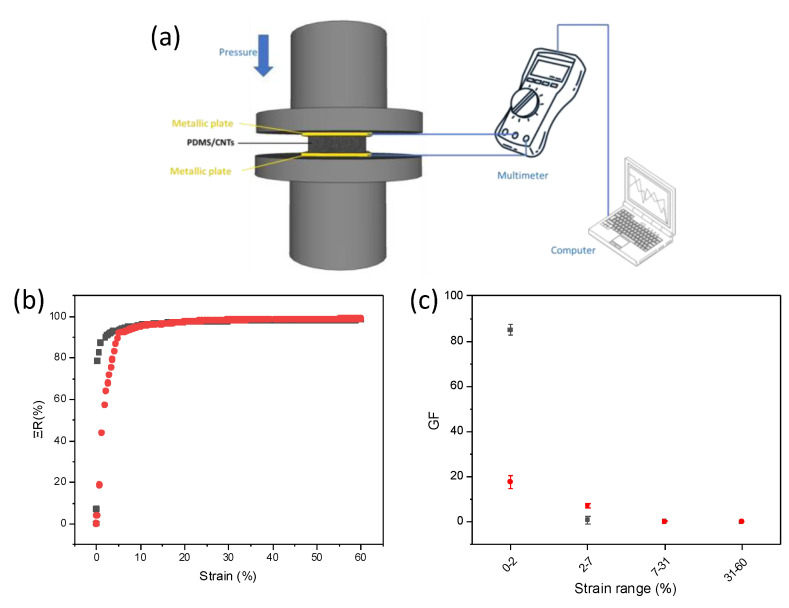
(**a**) Scheme of the set-up used for piezoresistive measurements, (**b**) relative resistance variation at different strain in PDMS/CNTs_long_ (gray curve), PDMS/CNTs_short_ (red curve), (**c**) gauge factor variation at different strains range for PDMS/CNTs_long_ (gray curve), PDMS/CNTs_short_ (red curve).

The piezoresistive behavior of the two different types of sponges were evaluated by recording the relative resistance variation (ΔR) as follows: ΔR = R − R_0_/R_0_, in which R_0_ and R are the resistance value at 0 and at any pressure, respectively. Figure 5a reports the experimental set-up for the piezoresistive measurements. In brief, the PDMS/CNTs foam was placed between two metallic plates, with an electrical wire welded onto each plate, and connected to a multimeter. The applied pressure was controlled using a dynamometer with two circular parallel tools, between which were placed the foam with the metallic plates. ΔR variations were evaluated until a 60% strain which is 5% higher with respect to the strain necessary to monitor a human joint movement. Interestingly, the ΔR values varied in all the analyzed range for all the foams (Figure 5b), suggesting both the nanocomposites can be used to monitor strain variations up to at least 60% strain. All the materials work as ohmic resistance under various compression strains (Figures S2 and S3). By evaluating the slope of the two curves in Figure 5b, the sponges evidenced different sensitivities in different strain ranges. To quantify and evaluate the differences among the materials, we calculated the gauge factor (GF) for the linear ranges of each curve. GF is a parameter that quantitatively expresses the sensitivity of the materials to strain, and is calculated as GF = (dΔR/R_0_)/(dΔL/L_0_) where L and L_0_ represent the length of the foams at any given strain value and at 0 strain. In particular, the curves can be approximated with four different linear ranges (R^2^ > 0.9) for each sponge. The obtained results are reported in Figure 5c. In the first range, between 0 and 2% strain, the PDMS/CNTs_long_ evidenced an outstanding GF (~85), which is five times higher with respect to the PDMS/CNTs_short_ foam. At higher strains, between 2 and 7%, the values were overturned, with PDMS/CNTs_short_ foam showing better performances. Finally, for strains higher than 7%, the two different foams evidenced the same behavior. Interestingly, the material evidenced a good reproducibility in all the tested ranges (Figure 5c; *n* = 3). It is evident that carbon nanotubes length affects the behavior of the piezoresistive material only at a lower strain. This could be due to the different mechanisms which are responsible for the change in resistance of the nanocomposite at different strains. Indeed, at lower strain the current variation is dominated by the formation of contact points among CNTs [20,33,34]. Therefore, at lower strains (<7%), the length of the nanomaterial dramatically affects this parameter, causing a variation in sensitivity values which appears to be favored by the presence of longer carbon nanotubes. Since resistance variation is monitored to determine the sensor sensitivity, the better performances of the nanocomposite prepared with longer CNTs can be explained by considering the obstacle effect of polymer chains on CNT junction gap variations, and is poorly affected by the initial conductivity of the material [35]. In fact, during the polymerization step, PDMS chains can be intercalated among the CNT network. Once pressure is applied on the piezoresistive materials, both the CNT network and the polymer chains are rearranged, and new electrical contacts between CNTs can occur, thus causing a change in electrical resistance at low strains. Intuitively, the establishment of a new contact point between two adjacent CNTs at lower strains (<2%) is more probable on longer nanomaterial, due to the increased surface area. This allows the creation of a better percolation network and a more marked change in electrical resistance at low strain with respect to that observed on shorter CNTs. For higher strains (>7%), the current variation in the foams is dominated by the changing in dimension of the pores [20,33,34]. Considering that the two materials evidenced the same pore distribution (see Figure S1), the sensitivity values are overlapped for both the nanocomposites, independently from the kind of the CNTs used. This also confirms the poor effect of nanomaterial conductivity on piezoresistive performance. It is important to highlight that the values recorded for all the pressures on both the sponges are one order of magnitude higher with respect to the other piezoresistive materials based on carbon/PDMS composites [36,37,38], and with comparable values obtained on the most-performing composites [34,39,40,41].

**Figure 6:**
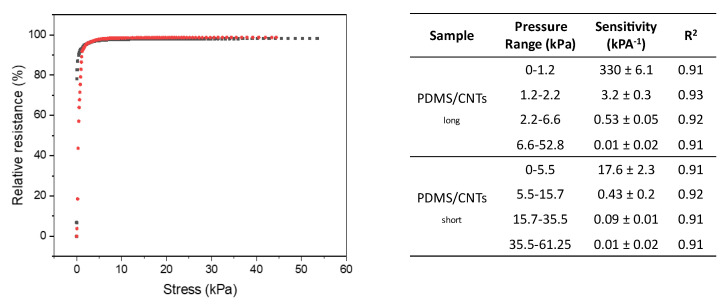
Relative resistance variation at different strains in PDMS/CNTs_long_ (gray curve) and PDMS/CNTs_short_ (red curve). The embedded table reports the recorded values observed for the different linear ranges observed in the two curves.

Although GF can give an idea of piezoresistive sponge performances and behavior, pressure sensitivity should also be evaluated in order to understand the effective capacity of piezoresistive materials to monitor low displacement under minimum forces. The pressure sensitivity (S) can be calculated as S = dΔR/R_0_/dP, in which P is the applied pressure value in pascals. From Figure 6, it is evident that the use of MWCNTS with different lengths allows the production of materials with different measurable pressure ranges. At the same time, an increment in low-pressure sensitivity is observed for the nanocomposites produced with longer MWCNTs, while these differences disappear at higher pressure. Further details shows that the experimentally recorded curves can be approximated with four linear fit (R^2^ > 0.9), as reported in the table embedded in Figure 6. The larger differences in sensitivities can be observed at lower pressure, since the current variations are dominated by the formation of new contact points between carbon nanotubes, analogously to what was observed in the evaluation of the GF. For pressures higher than 35 kPa, sensitivity did not vary between the two nanocomposites, because the current variation is dominated by the change of pore dimension. The only difference at higher pressure is represented by the increase in the pressure that can be sensed. In particular, longer carbon nanotubes allow the monitoring of higher pressures, due to the increase in mechanical stability of the nanocomposite. Interestingly, the values relative to pressure sensitivities recorded in all the materials prepared here are incredibly low with respect to other most-performing systems reported in the literature [33,36,40,42,43]. In particular, the PDMS/CNTs_long_ sponge showed an outstanding value of 330 kPa^−1^ for pressures lower than 1 kPa.

From the first linear fitting of Figure 6a, we calculated the minimum pressure that both the sponges can sense over the noise of 3.3σ_0_, in which σ_0_ represents the standard deviation of the relative resistance at zero strain. The PDMS/CNTs_short_ shows a good result, being able to monitor a value of compression of 1.8 Pa, which is significantly lower with respect to the other high-performing materials reported in the literature [33,36,40,42,43]. However, the use of longer MWCNTS allows the decrease of the LOD up to 0.2 Pa, confirming the importance of the morphology of MWCNTs in producing a piezoresistive sensor with improved performance.

**Figure 7:**
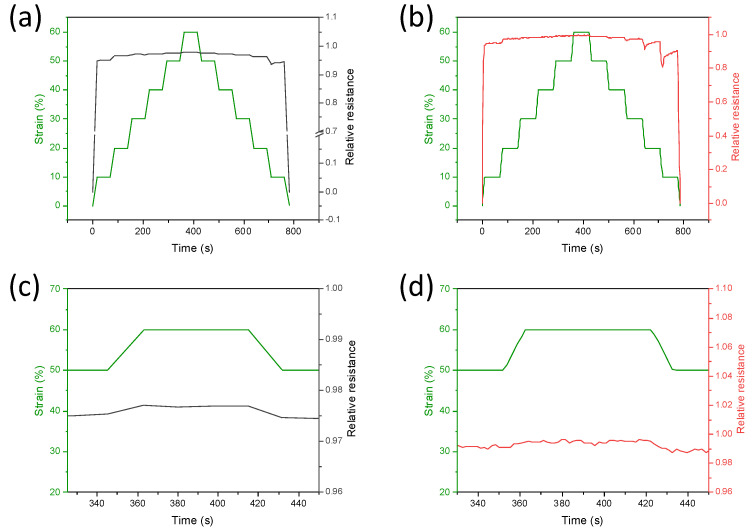
Time-resolved current variation at different strains measured on PDMS/CNTs_long_ (gray curve), (**a**) and PDMS/CNTs_short_ (red curve). (**b**) Sponges subjected to loading/unloading steps from 0 to 60%. In (**c**,**d**), the enlargement at 60% strain is reported.

The response time and stability of the PDMS composites depending on the kind of MWCNTs used, were also evaluated (Figure 7). The PDMS/CNTs_long_ and PDMS/CNTs_short_ sponges were compressed between 0 and 60% strain and vice versa, with 10% steps at a compression rate of 5 mm/min (Figure 7a). In all cases, during compression the relative resistance changes, together with the stress, without any significant delay, and the increment is higher at low strains, confirming the different sensitivities of the materials at different pressures. During the release of the sponges, despite the PDMS/CNTs_long_ returning relatively quickly to the initial state, this does not happen in the case of PDMS/CNTs_short_. However, applying a new cycle of compression, it is observable that the sponges composed of shorter carbon nanotubes completely recover their mechanical and electrical properties (Figure S4). This is evidence that, despite the fact that the mechanical stability of the PDMS/CNTs_short_ nanocomposites can be completely recovered in at least 100 s, shorter carbon nanotubes are not ideal for monitoring fast movements. Therefore, it can be deduced that the use of longer carbon nanotubes can allow the production of nanocomposites with better flexibility and mechanical and electrical stability.

**Figure 8:**
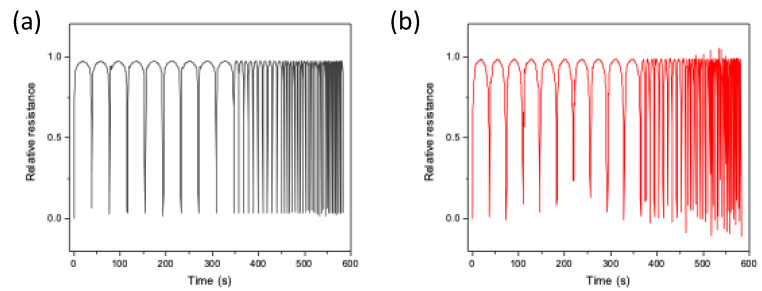
Dynamic piezoresistive properties of PDMS/CNTs_long_ (**a**) and PDMS/CNTs_short_ (**b**) sponges at strain rates of 5, 20, 35, 45 and 60 mm/min (10 cycles for each step) in the range 0–20%.

To further characterize the sponges, the stability under different cycles at 20% strain was also evaluated (Figure 8). Notably, the PDMS/CNTs sponge prepared using longer carbon nanotubes has a very good response, being able to follow all the compression and release cycle at different frequencies with good reproducibility. On the other hand, the electrical response of the PDMS/CNTs_short_ sponge appears more variable. This in part confirms what was already observed in Figure 7, thus suggesting that composites made of shorter carbon nanotubes need more time to recover their original state, thus causing a less reproducible response during fast compression/decompression cycles.

## 5. Conclusions

The present work explored for the first time the effect of MWCNTs length on mechanical and electrical properties of PDMS/CNTs sponges when used as piezoresistive sensors. This was possible because we used a solvent-free mechanochemistry procedure for MWCNTs dispersion, which did not require chemical functionalization, thus avoiding the modification of electronic and morphological properties of the nanomaterials. Despite this aspect has been never considered during the precedent development of porous piezoresistive materials, we observed as this is important to obtain sensors with improved performances. Our studies demonstrated as the use of shorter carbon nanotubes, not only cause a decrease in conductivity and mechanical stability of the materials, but also piezoresistive properties are negatively affected. In fact, when used as piezoresistive sensor, the material fabricated with longer carbon nanotubes evidenced better gauge factors, sensitivities at low strain and an outstanding limit of detection of 0.2 Pa, which is 9 times lower with respect to that recorded when shorter CNTs are used for the nanocomposite fabrication. Moreover, these values are significantly better than the most performing piezoresistive sensors already described in literature. Also, the response time is affected by CNTs length especially during the release of the material, in fact, despite both the materials are able to return to their original state after pressure/release cycles, the process is faster when longer CNTs are used. In conclusion this work highlight the importance of the morphological characteristics of the CNTs used as a filler in piezoresistive nanocomposites suggesting as should be avoided the use of shorter CNTs or chemical reactions that can cause the shortening of the nanomaterial (e.g., chemical oxidation).

## Data Availability

Not applicable.

## Supplementary Materials

The following supporting information can be downloaded at: https://www.mdpi.com/article/10.3390/nano12213741/s1, Figure S1: Pore size distribution of (a) PDMS/CNTs_long_ and (b) PDMS/CNTs_short_ foams; Figure S2: Current−voltage curves recorded at different strain levels from 10 to 60% on PDMS/CNTs_long_; Figure S3: Current−voltage curves recorded at different strain levels from 10 to 60% on PDMS/CNTs_short_; Figure S4: Two consecutive time-resolved current variations measured on PDMS/CNTs_short_ (red curve) sponge subjected to loading/unloading steps from 0 to 60%.
