# Supplementary material for: Does Size Matter? The Case of Piezoresistive Properties of Carbon Nanotubes/Elastomer Nanocomposite Synthesized through Mechanochemistry
Source: Nanomaterials (Basel). 2022 Oct 25;12(21):3741. doi: 10.3390/nano12213741 (PMC9658284; doi:10.3390/nano12213741)
Supplement: Supplementary file 1 [file nanomaterials-12-03741-s001.zip › nanomaterials-1986066-Supplementary.pdf]

Does size matter? The case of piezoresistive properties of carbon nanotubes/elastomer nanocomposite synthesized through mechanochemistry

Antonio Turco[a]*, Anna Grazia Monteduro[a,b], Francesco Montagna[c], Elisabetta Primiceri[a], Mariaenrica Frigione[c] and Giuseppe Maruccio[a,b]*

[a] CNR Nanotec Institute of Nanotechnology, Via Monteroni, 73100 Lecce, Italy.

[b] Department of Mathematics and Physics “Ennio De Giorgi”, University of Salento, Omnics Research Group, Via per Monteroni, 73100 Lecce, Italy

[c] Department of Innovation Engineering, University of Salento, Prov.le Lecce-Monteroni, 73100 Lecce, Italy

Corresponding author: antonio.turco@nanotec.cnr.it; giuseppe.maruccio@unisalento.it

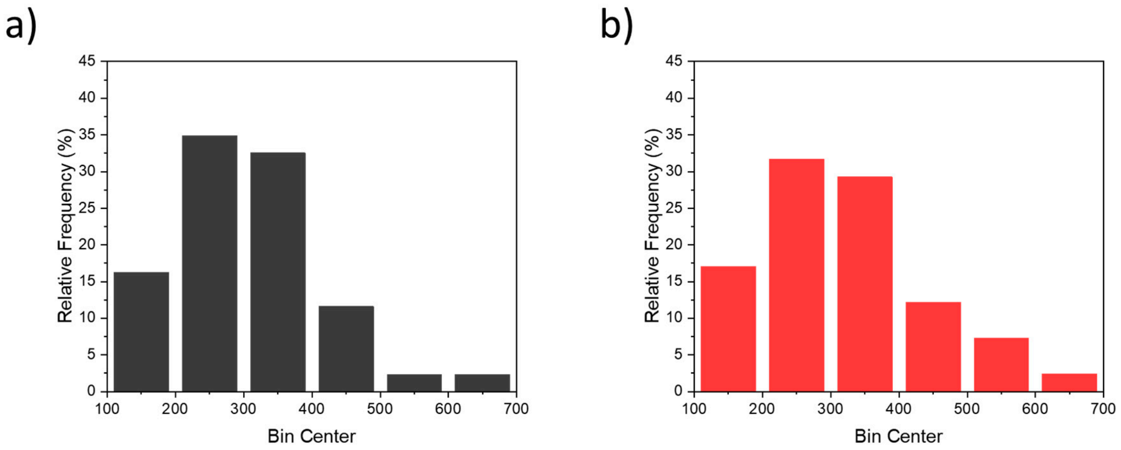

Figure S1. Pore size distribution of (a) PDMS/CNTs$_{long}$ and (b) PDMS/CNTs$_{short}$ foams.

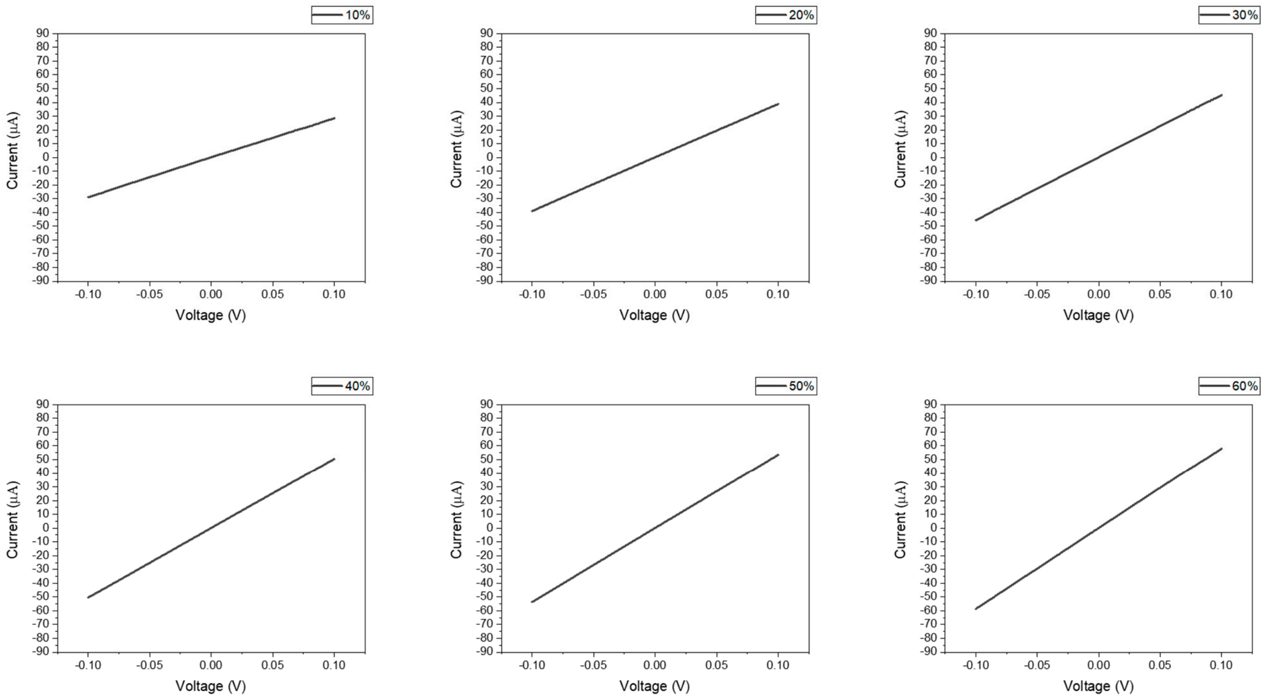

Figure S2. Current–voltage curves recorded at different strain levels from 10 to 60% on PDMS/CNTs$_{long}$.

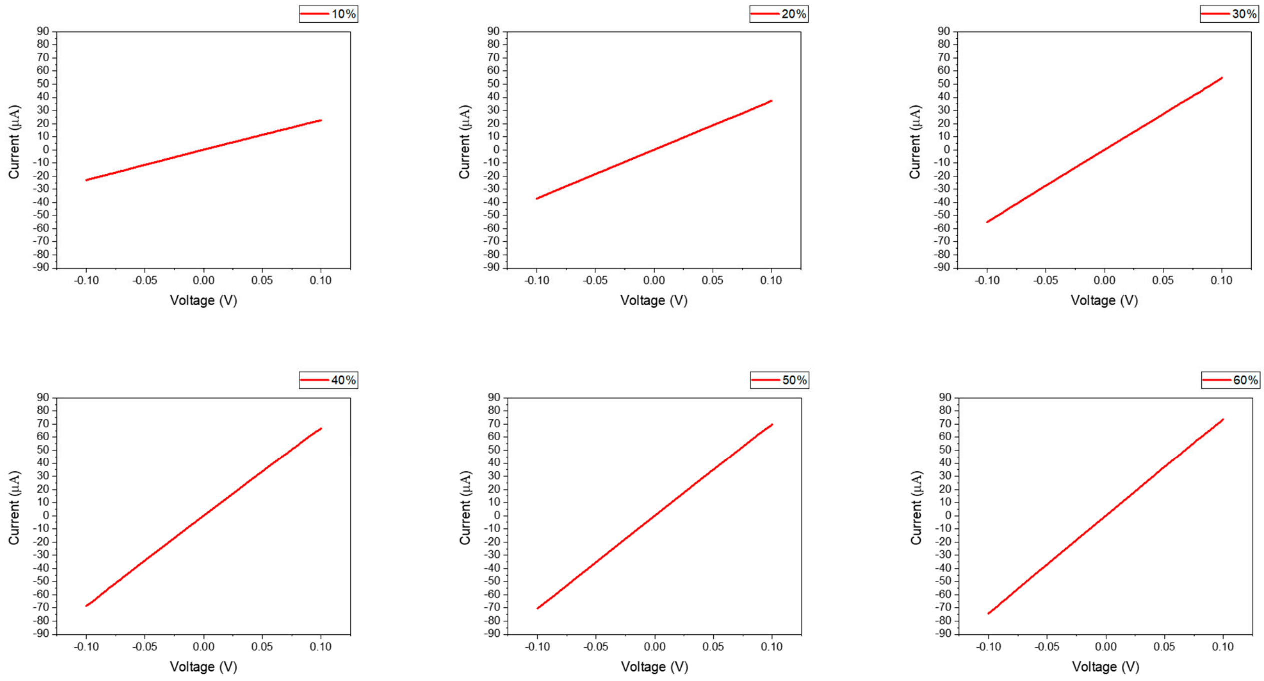

Figure S3. Current–voltage curves recorded at different strain levels from 10 to 60% on PDMS/CNTs$_{short}$.

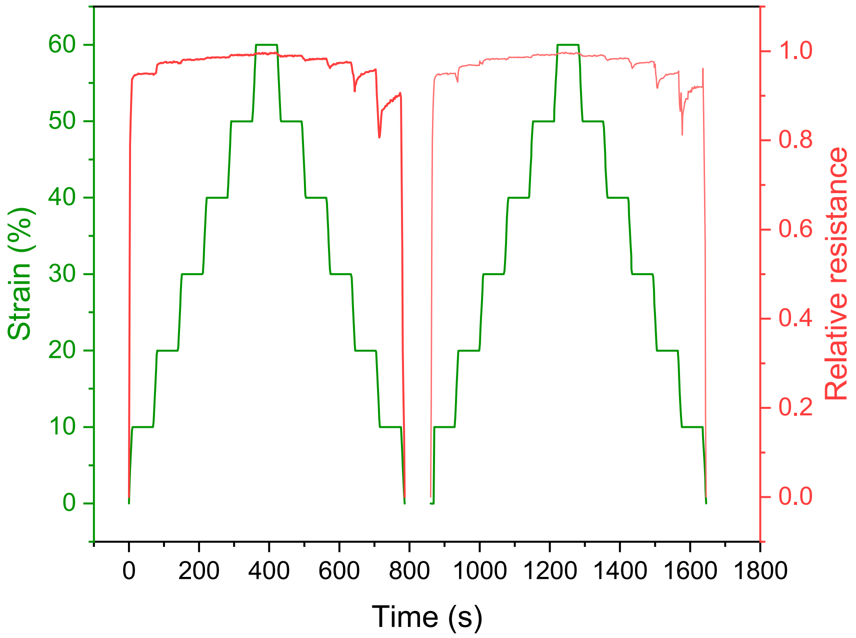

Figure S4. Two consecutive time-resolved current variation measured on PDMS/CNTs$_{short}$ (red curve) sponge subjected to loading/unloading steps from 0 to 60%.
